# Preclinical evaluation of bacterially produced RSV-G protein vaccine: Strong protection against RSV challenge in cotton rat model

**DOI:** 10.1038/srep42428

**Published:** 2017-02-10

**Authors:** Sandra Fuentes, Laura Klenow, Hana Golding, Surender Khurana

**Affiliations:** 1 Division of Viral Products, Center for Biologics Evaluation and Research (CBER), FDA, Silver Spring, MD, 20903, USA

## Abstract

In current study, we evaluated the safety and protective efficacy of recombinant unglycosylated RSV G protein ectodomain produced in *E. coli* (in presence and absence of oil-in-water adjuvant) in a preclinical RSV susceptible cotton rat challenge model compared to formaldehyde inactivated RSV (FI-RSV) and live RSV experimental infection. The adjuvanted G protein vaccine induced robust neutralization antibody responses comparable to those generated by live RSV infection. Importantly, adjuvanted G protein significantly reduced viral loads in both the lungs and nose at early time points following viral challenge. Antibody kinetics determined by Surface Plasmon Resonance showed that adjuvanted G generated 10-fold higher G-binding antibodies compared to non-adjvuanted G vaccine and live RSV infection, which correlated strongly with both neutralization titers and viral load titers in the nose and lungs post-viral challenge. Antibody diversity analysis revealed immunodominant antigenic sites in the N- and C-termini of the RSV-G protein, that were boosted >10-fold by adjuvant and inversely correlated with viral load titers. Enhanced lung pathology was observed only in animals vaccinated with FI-RSV, but not in animals vaccinated with unadjuvanted or adjuvanted RSV-G vaccine after viral challenge. The bacterially produced unglycosylated G protein could be developed as a protective vaccine against RSV disease.

RSV vaccine development efforts have been steadily increasing in recent years^1^,^2^ in order to reduce the incidence of RSV associated hospitalization and death resulting from acute lower respiratory infection (ALRI) in the first year of life among infants^3^,^4^. This could be achieved through either maternal or infant immunization, wherein, vaccine safety is of prime importance. The elderly are another potential target population for RSV vaccination due to significant increase in morbidity following repeat RSV infections^5^,^6^,^7^.

We recently demonstrated that primary RSV infection primarily results in increase in anti-RSV-G antibodies and the response to F and G proteins following natural infection are unlinked^8^. Specifically, while the titers and diversity of anti-F antibody response increased steadily with age, a significant decline in anti-G antibody titers was observed with increased age from infants to adults^8^. Therefore, both F and G proteins should be included in RSV vaccine candidates. To that end, in an earlier study, we evaluated the safety and protective activity of unglycosylated, bacterially produced RSV-A2 G protein in *E. coli* (REG; Recombinant *E. coli* produced G) in comparison with fully glycosylated G produced in mammalian cells (RMG; Recombinant Mammalian cell derived G) in a mouse model^9^. Neutralizing antibodies and complete reduction of lung viral loads after homologous (RSV-A2) and heterologous (RSV-B1) viral challenges were observed in animals vaccinated with REG, but not in RMG-vaccinated animals. Furthermore, enhanced lung pathology and elevated Th2 cytokines and chemokines were observed exclusively in animals vaccinated with RMG, but not with REG after homologous or heterologous RSV challenge^9^.

Cotton rats are more permissive to RSV infection than BALB/c mice. Consequently, the Cotton rat is considered a more relevant animal model than the mouse for preclinical studies on RSV pathogenesis, anti-RSV drugs, and RSV vaccine efficacy and safety^10^,^11^.

Therefore, the cotton rat model was used for pre-clinical evaluation of unglycosylated recombinant *E. coli* produced G protein (REG) as a potential RSV vaccine. We also examined the impact of adjuvant on immune response to REG and *in vivo* protection from RSV challenge. The adjuvant used in the current study, Emulsigen, is an oil-in-water adjuvant commonly used in veterinary vaccines. It is similar to adjuvants used in human clinical trials, such as MF59 and AS03^12^,^13^,^14^. We also included a group of animals that received FI-RSV vaccine lot #100, which was associated with enhanced lung pathology in young children and cotton rats following RSV infection^15^,^16^,^17^,^18^,^19^. Animals were challenged with RSV-A2 and were evaluated for viral loads in both lungs and nasal homogenates on days 2 and 5 post challenge as well as for lung pathology as part of risk assessment.

## Results

### Neutralizing antibody response following immunization of female cotton rats with RSV-G protein, FI-RSV and live RSV experimental infection

**Figure 1:**
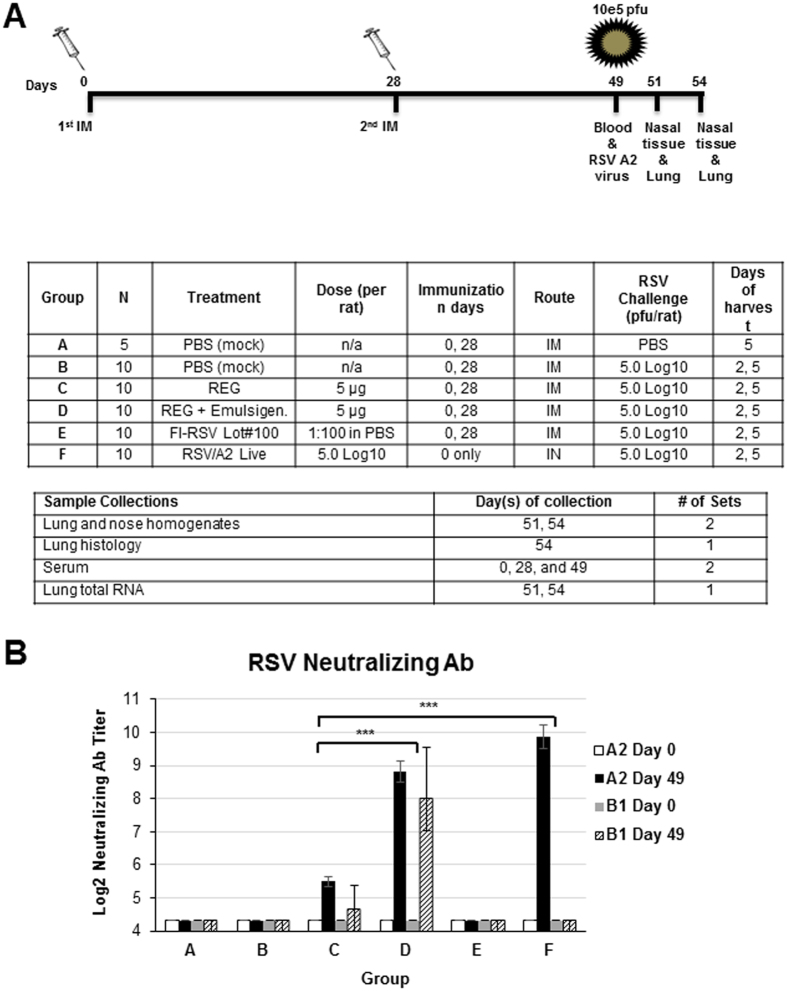
Neutralizing antibody response following RSV-G protein, FI-RSV and live RSV experimental infection. (**A**) Schematic representation of cotton rat immunization and challenge schedule. Inbred female *Sigmodon hispidus* cotton rats between 6 and 8 weeks of age were immunized i.m. with PBS (Gps A-B), 5 μg of unadjuvanted or Emulsigen-adjuvanted REG (Recombinant *E. coli* produced G) (Gps C-D), or with FI-RSV (Gp E) on days 0 and 28 in groups A thru E, or were infected intranasally (i.n.) with 0.1 ml of RSV/A2 at 10^5^ pfu per rat (Gp F). Blood was collected by eye-bleed on days 0, 28 and 49. On day 49, animals were either mock challenge intranasally with 0.1 ml of PBS (Gp A), or with 0.1 ml of RSV-A2 virus at 10^5^ pfu per animal (10 animals per group) (Gps B-F). Cotton rats were sacrificed on days 2 or 5 post-challenge wherein lungs and nose tissues were collected. (**B**) Sera from individual cotton rats collected at pre-vaccination (day 0) and 3 weeks post second immunization (day 49) were tested for neutralization in a plaque reduction neutralization test (PRNT) against the homologous RSV-A2 strain and heterologous RSV-B1 strain. Neutralizing antibody titers represent 50% inhibition of plaque numbers. Statistical significance was tested with one-way ANOVA and Bonferroni multiple comparisons tests. ***p < 0.0001.

**Figure 2:**
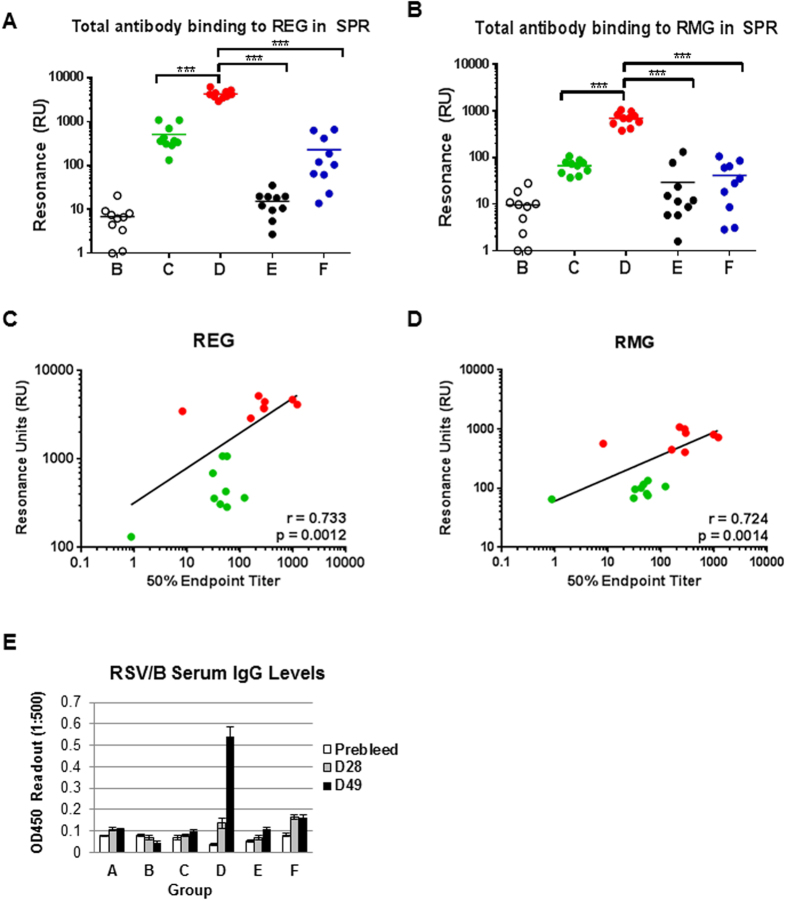
Surface Plasmon Resonance analysis of post-vaccination cotton rat serum antibody titers against REG and RMG and correlations with PRNT neutralization titers. (**A**,**B**) The same individual post-vaccination cotton rat sera from Fig. 1 were tested for total antibody binding to the *E.coli* produced unglycosylated RSV-G protein (REG; Recombinant *E. coli* produced G) protein (**A**) or glycosylated RSV-G (RMG; Recombinant Mammalian cell derived G) protein (**B**) by Surface Plasmon Resonance (SPR). Total antibody binding is represented as SPR resonance units. Statistical significance was tested with one-way ANOVA and Bonferroni multiple comparisons tests. ***p < 0.0001, **p < 0.001, *p < 0.05. (**C**,**D**) Relationship between the total anti-G antibody binding to REG (**C**) or RMG (**D**) of each individual post-REG vaccination sera (groups C and D) from immunized animals in SPR (shown in Fig. 2A and B for binding to REG and RMG respectively) and post-vaccination PRNT neutralization titers (shown in Fig. 1A) was analyzed with Spearman’s correlations. (**E**) ELISA binding of post-vaccination cotton rat serum antibodies against RSV B1 virus.

Cotton rats have been established as a relevant animal model for preclinical studies of RSV infection, evaluation of therapeutics, vaccine-induced protection or vaccine associated enhanced respiratory disease (VAERD)^19^,^20^,^21^,^22^. Therefore, we used this animal model for preclinical evaluation of bacterially produced G protein as a candidate RSV vaccine. As outlined in Fig. 1A, 6 to 8 weeks old inbred female *Sigmodon hispidus* cotton rats were immunized intramuscularly (i.m.) twice with PBS (groups A-B), with 5 μg of unadjuvanted (group C) or Emulsigen-adjuvanted RSV G (group D), or with FI-RSV (lot #100) (group E), on days 0 and 28, or were infected once intranasally (i.n.) with 0.1 ml of live RSV-A2 at 10^5^ pfu per rat (group F). On day 49, animals were either mock challenged intranasally (i.n.) with 0.1 ml of PBS (group A), or with 0.1 ml of RSV-A2 virus at 10^5^ pfu per animal (groups B-F). Serum samples from individual cotton rats collected at pre-vaccination (day 0) and 3 weeks post second immunization (day 49) were tested for neutralization in a plaque reduction neutralization test (PRNT) against the homologous RSV-A2 strain. As shown in Fig. 1B, the positive control Gp F (infected with live RSV-A2) demonstrated high neutralizing antibody titer (9–10 log2). In contrast, the FI-RSV vaccinated animals did not generate neutralizing antibodies, similar to the PBS immunized negative control animals (Gps A and B). The unadjuvanted REG protein generated weak neutralization titers (Fig. 1B Gp C), while cotton rats vaccinated with REG mixed with Emulsigen generated high neutralization titers (8–9 log2) that were comparable to live RSV experimentally infected animals (Fig. 1B Gp D). In addition, only serum samples from group D (REG + Emulsigen) showed cross-neutralization of RSV B1, with titers ranging between 81.6–1280 (mean ± SD: 723.4 ± 533.6). The lack of RSV B1 neutralization with sera from experimentally infected animals (Gp F) was puzzling. However, it correlated with very low serum antibody binding to RSV B1 virions in ELISA (Fig. 2E).

### Surface Plasmon Resonance real-time antibody kinetic analysis of post-vaccination cotton rat sera to REG and RMG and correlation with neutralization titers

In addition to *in vitro* RSV-neutralizing antibodies, especially in the case of anti-G, some non-neutralizing G-binding antibodies have shown protective activity *in vivo*^22^,^23^,^24^,^25^. Therefore, all post-vaccination cotton rat sera were tested individually for antibody binding to the *E.coli* produced RSV G (REG) protein (Fig. 2A) or to a mammalian cell derived RSV G (RMG) protein that mimics the native fully glycosylated G protein on the viral surface (Fig. 2B) by Surface Plasmon Resonance (SPR). The sera from PBS-vaccinated animals gave background binding to both unglycosylated and glycosylated G proteins (Gp B; Maximum RU signal < 20). The FI-RSV vaccinated sera showed very low antibody binding (<100 RU) to either REG or RMG. Interestingly, sera from live RSV-A2 infected animals showed low binding to glycosylated RMG (Fig. 2B), and somewhat higher binding to unglycosylated REG (Fig. 2A). The unadjuvanted REG protein immunized animals elicited significant titers of binding antibodies against the unglycosylated REG and glycosylated RMG (Fig. 2A,B, Gp C). The Emulsigen-adjuvanted REG vaccine generated 10-fold higher antibody binding titers to both REG and RMG compared with the unadjuvanted REG (Fig. 2A,B, Gp D vs. C). For the REG vaccinated animals (groups C and D) the total antibody binding to either REG (Fig. 2C) or RMG (Fig. 2D) correlated strongly with neutralization titers for individual animals as measured by PRNT (*r = 0.733* and *r = 0.724*, respectively). These data suggested that the adjuvanted REG vaccine can generate high titer antibodies that bind to the native glycosylated G, in agreement with the virus neutralization data in Fig. 1B. To evaluate cross-reactivity of post-vaccination serum antibodies against RSV B1 virus, we measured binding of post-vaccination sera to RSV B1 virions using ELISA (Fig. 2E). Surprisingly, sera from experimentally infected cotton rats (Gp F, D28 and D49) showed low binding to RSV B1 virions, suggesting a highly homotypic response. However, the Emulsigen adjuvanted REG-vaccinated animals generated the most significant binding to RSV B1 on day 49 post immunization (Fig. 2E).

### RSV challenge of vaccinated cotton rats

To investigate the pathology of RSV virus infection on upper and lower respiratory tract in vaccinated cotton rats, nasal and lung tissues were harvested at two or five days following virus challenge from 5 animals each and processed for multiple tests as outlined in Fig. 1A. Because of limitation in animal availability, and to achieve statistical significance, all the groups were challenge with RSV A2 in the current study.

**Figure 3:**
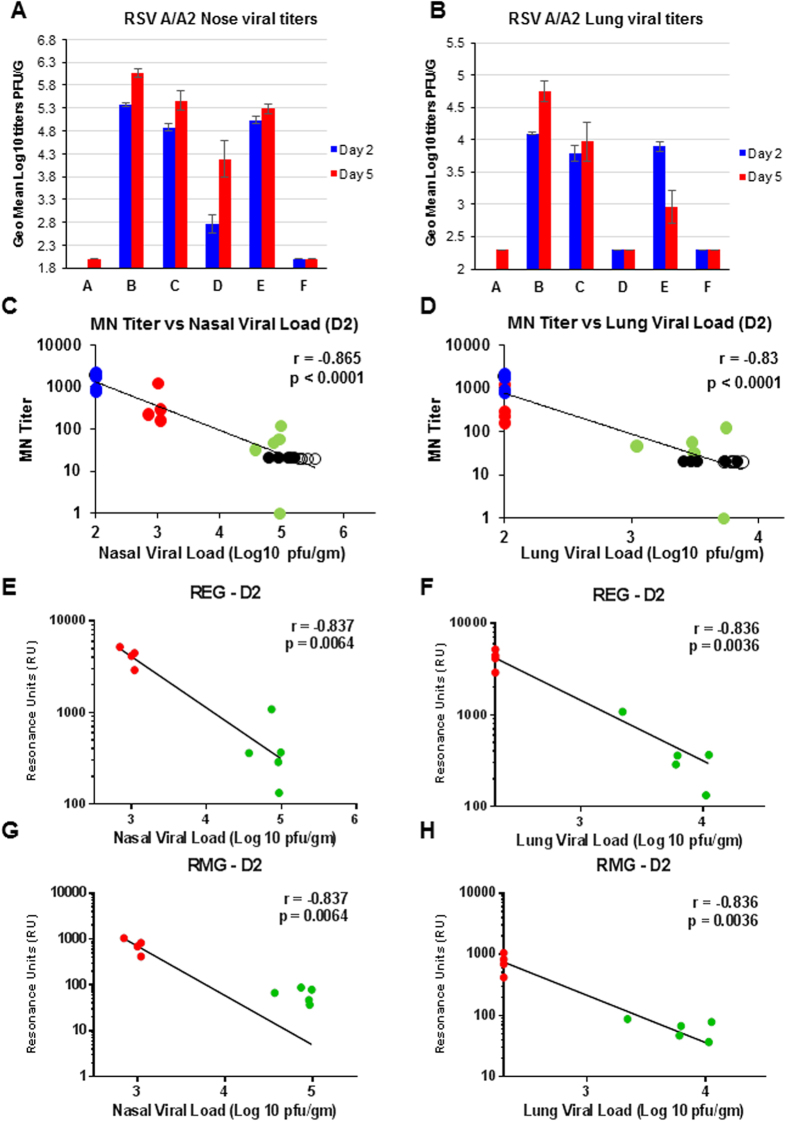
Immunization with adjuvanted REG protects cotton rats against RSV challenge. Cotton rats were challenged i.n. with 1 × 10^5^ pfu of RSV-A2 at 21 days after the second immunization. Two days post virus challenge, nose (**A**) and lung (**B**) tissues were collected and homogenized as described in materials and methods, and viral loads were determined by plaque assay. (**C**,**D**) Relationships between the PRNT neutralization titers in each individual post- vaccination sera from all vaccinated-viral challenged animals (Gps B-F) and either nasal viral loads (**C**) or lung viral loads (**D**) 2 days post-challenge with RSV-A2 with Spearman’s correlations. (E-H) Relationships between the total anti-G antibody binding to REG (**E**,**F**) or RMG (**G**,**H**) of individual post- vaccination sera from REG-vaccinated animals (Gps C and D) in SPR and nasal viral load (**E** and **G**) or lung viral load (**F** and **H**) at 2 days post-challenge with RSV-A2 with Spearman’s correlations. Total antibody binding to REG or RMG protein is represented in resonance units and viral loads are represented as pfu per gram tissue weight. The symbol colors correspond to the groups shown in Fig. 2.

RSV viral loads in the nose of cotton rats were evaluated on 2 and 5 days after intranasal RSV-A2 challenge (Fig. 3A). The highest nasal viral loads were measured in the PBS-immunized animals (Gp B; 5.4 and 6.1 Log10 PFU/g on days 2 and 5, respectively). No virus was detected in animals that were experimentally infected with live RSV-A2 (Gp F), suggesting a sterilizing immunity in this group. The FI-RSV immunized animals (Gp E) showed minimal reduction in nasal viral loads following viral challenge. Animals immunized with unadjuvanted REG protein (Gp C) showed ~0.5 Log10 reduction in nasal viral load on days 2 and 5 post RSV-A2 infection compared to mock-immunized, RSV-challenged animals (Gp B). In contrast, animals immunized with adjuvanted REG vaccine (Gp D) had significantly reduced viral load in the nose compared to the control (Gp B) on both days 2 and 5 post-infection (Fig. 3B).

Highest lung viral load were detected in animals mock-immunized with PBS and infected with RSV (Fig. 3B Gp B; 4.1 and 4.8 Log10 PFU/g on days 2 and 5, respectively). In contrast, no virus was detected in animals that were experimentally infected intranasally with RSV-A2 (Gp F). In FI-RSV-immunized animals a high level of viral replication was detected on day 2 that declined by day 5 after RSV-A2 challenge (Gp E). Animals immunized with unadjuvanted REG protein (Gp C) had slightly reduced pulmonary viral loads compared to mock-immunized RSV-A2 challenged animals (~0.8 Log10 reduction on day 5). However, animals immunized with adjuvanted REG protein (Gp D) had significantly reduced pulmonary viral load compared to Gp B controls, with almost undetectable viral replication in the lung on both day 2 and 5 post viral challenge. The reduction in lung viral loads in the adjuvanted-REG vaccine group was identical to the RSV-A2 infection (positive control) (Gp F).

### Correlates of protection from nasal and lung viral replication following RSV challenge in cotton rats

It was important to determine the relationship between reductions in viral load post RSV-A2 challenge *in vivo* with the *in vitro* antibody assays following vaccination. As can be seen in Fig. 3, a strong inverse correlation was observed between the viral loads at day 2 post viral challenge in nose (Fig. 3C) or lungs (Fig. 3D) and the PRNT titers for all vaccinated groups.

We also correlated nasal and lung viral loads in the REG vaccinated animals (Gps C and D) with total anti-G antibody binding (Maximum RU) to either the unglycosylated REG (Fig. 3E and F) protein or to fully glycosylated G (RMG) protein (Fig. 3G and H). In all analyses a strong inverse correlation was found between viral loads at day 2 post RSV-A2 challenge of individual animals and post-vaccination serum antibody binding to both the unglycosylated or glycosylated forms of G protein in SPR.

**Figure 4:**
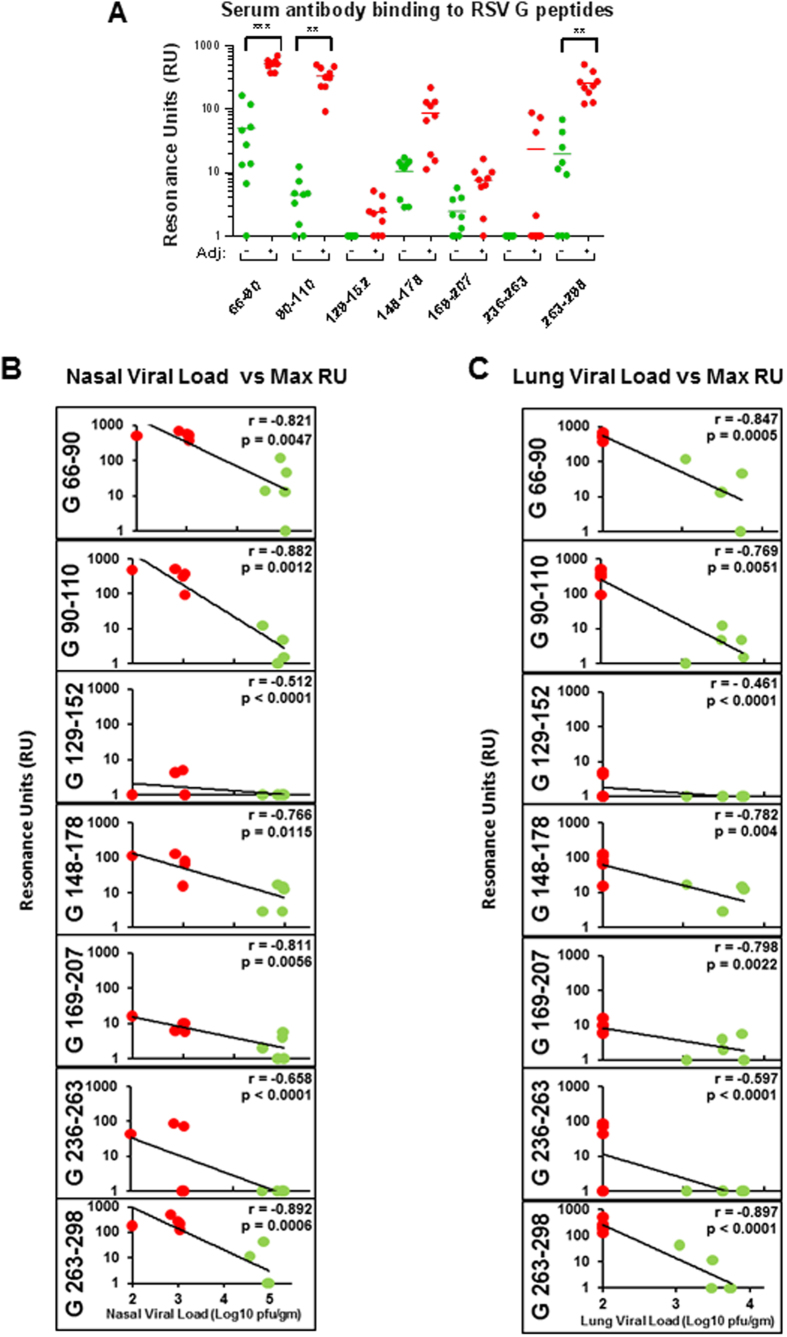
Diversity of post-REG vaccination antibody response against different antigenic sites within RSV G and correlation with nasal and lung viral loads following RSV challenge. All individual post-REG vaccination cotton rat sera (groups C and D) were tested for total antibody binding to the biotinylated peptides representing different antigenic sites within the extracellular domain of RSV-G consisting of amino acids 66–90, 90–110, 129–152, 148–178, 169–207, 236–263 and 263–298 of the RSV G protein using real time SPR kinetics (A). Total antibody binding is represented as SPR resonance units. Statistical significance was tested with one-way ANOVA and Bonferroni multiple comparisons tests. ***p < 0.0001, **p < 0.001, *p < 0.05. (**B**,**C**) Relationship between the total antibody binding of each individual post- vaccination sera from immunized animals against different antigenic sites within the extracellular domain of RSV-G representing amino acids 66–90, 90–110, 129–152, 148–178, 169–207, 236–263 and 263–298 and either nasal viral loads (**B**) or lung viral loads (**C**) in cotton rats at 2 days post-challenge with RSV-A2 with Spearman’s correlations. Total antibody binding is represented as SPR resonance units and lung viral loads are represented as pfu per gram tissue weight.

The diversity of REG generated post-vaccination polyclonal serum antibodies were further explored using biotinylated peptides captured on SPR chip surface representing different antigenic sites within the extracellular domain of RSV-G consisting of amino acid residues 66–90, 90–110, 129–152, 148–178, 169–207, 236–263 and 263–298 of the RSV G protein (Fig. 4A) identified previously in the post-infection human sera^8^. Interestingly, the peptide containing central conserved domain (CCD) motif (aa 169–207) was only moderately recognized by the REG vaccinated cotton rat sera (either adjuvanted or unadjuvanted groups). An antigenic site at the amino terminus of the CCD motif represented by the peptide 148–178, which contains the epitope of the protective monoclonal antibody 131–2G^25^,^26^, was recognized by all unadjuvanted and adjuvanted REG vaccinated sera. In addition, strong antibody binding was observed to antigenic sites located in the N- and C-termini of the RSV G protein. In all cases, antibody binding of polyclonal sera from the animals vaccinated with the adjuvanted-REG protein were 10–100 fold higher compared with the unadjuvanted-REG immune sera (Fig. 4A, green vs. red circles) against different antigenic sites within RSV G protein. However, these differences reached statistical significance only for the two N-terminal (peptides 66–90 and 90–110) and the C-terminus (peptide 263–298) antigenic sites. Binding to peptides 129–152 and 169–207 was also higher for the adjuvanted group, but due to intra group variability did not reach statistical significance (p > 0.05).

The levels of antibody binding to the different antigenic sites within the extracellular domain of RSV G protein for REG vaccinated animals in Gps C and D (shown in Fig. 4A) were inversely correlated with nasal and lung viral loads on 2 days post-challenge with RSV-A2 (Fig. 4B and C), except peptide 129–152. Therefore, the REG vaccine elicited a diverse anti-G antibody repertoire in cotton rats that may contribute to *in vivo* control of virus replication after challenge with the RSV-A2 virus. Unadjuvanted REG elicited lower and more heterogeneous binding titers, while the adjuvanted REG generated significantly higher antibody titers with lower variability among animals that correlated with better control of viral replication in both the upper and lower respiratory tract following RSV-A2 viral challenge.

### Histopathology of lungs from vaccinated cotton rats following virus challenge

One of the main concerns with any RSV vaccine is the potential for vaccine associated enhanced respiratory disease (VAERD) as represented by lung pathology and lung cytokines^19^,^20^,^21^,^27^. In addition to FI-RSV vaccine that was used in clinical trials, subsequent studies with recombinant F and G proteins also demonstrated VAERD in animal models^23^,^28^,^29^. Therefore, it was important to determine if the REG vaccination resulted in differential lung pathology after RSV-A2 viral challenge in this preclinical study.

**Figure 5:**
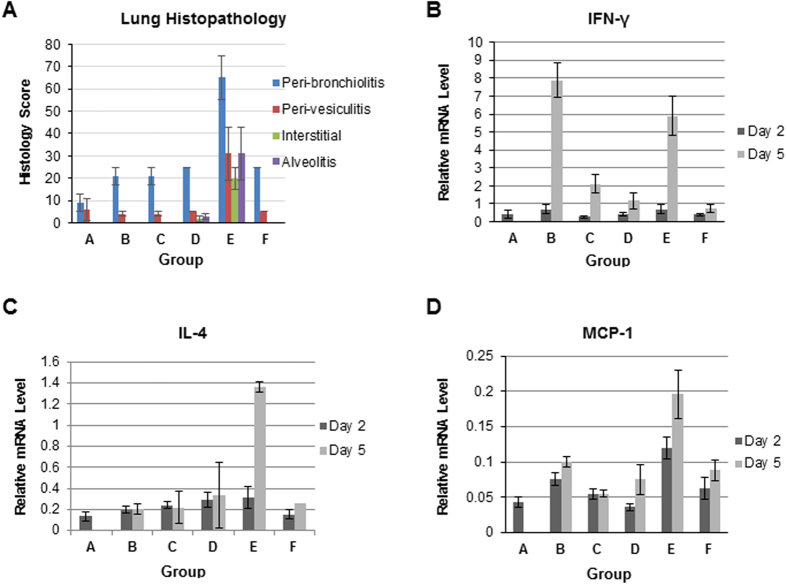
Histopathology and cytokine/chemokine mRNA analyses in lungs from vaccinated cotton rats following RSV challenge. (**A**) Lung tissues were collected at 5 days post-challenge with RSV-A2 and were stained with hematoxylin and eosin. Individual lungs were scored for pulmonary inflammation: peribronchiolitis (inflammatory cell infiltration around the bronchioles), perivasculitis (inflammatory cell infiltration around the small blood vessels), interstitial pneumonia (inflammatory cell infiltration and thickening of alveolar walls), and alveolitis (cells within the alveolar spaces). Slides were scored blindly using a 0–4 severity scale. The scores were subsequently converted to a 0–100% histopathology scales. (**B**–**D**) Cytokines and chemokines mRNA in lung homogenates from day 2 and day 5 post viral challenge were measured by RT-qPCR using primers specific for IFN-γ (**B**), IL-4 (**C**) or MCP-1 (**D**). The baseline cycles and cycle threshold (Ct) were calculated by the iQ5 software in the PCR Base Line Subtracted Curve Fit mode. The Ct values were plotted against Log10 cDNA dilution factor. These curves were used to convert the Ct values obtained for different samples to relative expression units. These relative expression units were then normalized to the level of β- actin mRNA (“housekeeping gene”) expressed in the corresponding sample. For animal studies, mRNA levels are expressed as the geometric mean ± SEM for all animals in a group at a given time.

Lungs collected at 5 days post-challenge with RSV-A2 virus from vaccinated cotton rats were scored for pulmonary inflammation: peribronchiolitis, perivasculitis, interstitial pneumonia and alveolitis (Fig. 5A). The only animals with increased lung pathology as evident by elevated histopathology scores were those vaccinated with FI-RSV (Gp E). Importantly, the animals vaccinated with either unadjuvanted or adjuvanted REG (Gps C and D) had comparable histology scores to the PBS-mock (Gp B) or live RSV experimentally infected animals (Gp F).

### Lung cytokine expression levels measured by qRT-PCR

The increase in Th2 cytokines and chemokines was a hallmark of VAERD following FI-RSV vaccination in previous studies^21^,^30^. Unfortunately, due to absence of appropriate reagents to measure cytokine proteins in cotton rats, it is not possible to establish IL4/IFN-γ protein ratios. Instead, pulmonary expression of several cytokines (IL-4, IL-2, IFN-γ, MIP-1α, and MCP-1) mRNA was evaluated in the lung tissues of cotton rats collected on days 2 and 5 after RSV infection by qRT-PCR. While expression of IFN-γ mRNA was strong in mock-vaccinated animals (Group B) and in animals vaccinated with FI-RSV (Fig. 5B; Gp E) after viral challenge, expression of IL-4 and MCP-1 mRNAs was highest in FI-RSV vaccinated animals (Fig. 5C,D; Gp E). Importantly, there was no evidence of increase in IL-4 or chemokines mRNAs in the lungs of cotton rats vaccinated with either adjuvanted REG (complete protection) or unadjuvanted REG (incomplete protection) after RSV-A2 viral challenge. These findings are in agreement with our study in the mouse model^9^. These data support the safety of the recombinant bacterially expressed G protein in the cotton rat model in the preclinical study.

## Discussion

This preclinical study in the cotton rat model demonstrated that unglycosylated RSV G protein (REG) vaccine generated antibodies that bound to both unglycosylated and glycosylated forms of RSV G protein and modest virus-neutralizing titers that were significantly boosted by combining it with oil-in-water (Emulsigen) adjuvant. Furthermore, the adjuvanted REG generated antibodies that bound to RSV B1 virions and neutralized RSV B1 in PRNT. The specificity of the antibodies elicited by adjuvanted REG was explored by SPR and demonstrated broad diversity of epitopes spanning the entire RSV G ectodomain. It is likely the oil-in-water adjuvant boosted the innate responses and increased the number of CD4+ T-helper cells required for a robust antibody responses in naïve animals as previously demonstrated for other oil-in-water adjuvants^31^,^32^,^33^.

Reduction in lung and nasal viral loads was most efficient in the group experimentally infected with live RSV-A2 intranasal infection. These animals had high virus neutralization titers. Based on the SPR data, the majority of antibodies in this group targeted the F protein, since binding to RMG was very low (Fig. 2B). These findings are in agreement with previous study, wherein robust RSV F-specific titers were observed following primary RSV A2 infection with minimal responses against the RSV G protein^34^. Surprisingly, we did not observe cross reactivity with RSV B1 virions and no neutralization of RSV B1 in spite of high conservation in the F protein among RSV types. Therefore, natural infection in different species may result in different immune dominance patterns, and may also be influenced by genetic factors. In addition to strong antibody responses, live RSV infection could elicit cytotoxic T cells against both membrane and internal RSV proteins that could help in viral clearance in the upper and lower respiratory tract^35^,^36^.

Animals vaccinated intramuscularly with adjuvanted REG showed complete reduction in lung viral loads on both day 2 and day 5 post viral challenge. Reduction of viral replication in the nasal cavity was partial in adjuvanted-REG vaccinated cotton rats compared to PBS vaccinated animals. This finding is in agreement with earlier studies of passive immunization using IVIG and RSV-IG in children and cotton rats, which also demonstrated little or modest reduction of RSV replication in nasal tissues, perhaps because of limited penetration of systemic IgG into the nasal cavity^22^,^37^.

We explored the correlate of protection following vaccination with REG in this preclinical study. A strong negative correlation was observed between *in vitro* post-vaccination serum virus neutralization titers and viral loads in both the upper respiratory tract (nose) as well as lower respiratory tract (lungs) following viral challenge. In addition, statistically significant negative correlation was observed between post RSV-A2 challenge viral loads and total antibody binding to both unglycosylated REG and fully glycosylated RMG, predicting good binding to virus associated native G protein. It is very likely that not all anti-G protective antibodies can be measured in the *in vitro* PRNT assay, as previously demonstrated for the *in vivo* protective Mab 131–2G (independent of Fc function) that is non-neutralizing in classical PRNT assay^25^,^26^.

The specificity of the antibodies generated by the adjuvanted REG in the cotton rats were similar to those obtained in REG-vaccinated mice with some differences observed in epitope immune-dominance^9^. The mouse immune sera reacted equally well to all the G-derived peptides, while in the case of cotton rats, higher binding was measured against N-terminus peptides (66–90 and 90–110) and C-terminus peptide (263–298). Still, peptide 148–178, partially overlapping with the CCD domain, was also recognized by all sera from REG vaccinated animals (either unadjuvanted or adjuvanted). This modest species difference may reflect differences in B or T cell repertoires as well as HLA presentation of T cell epitopes. Importantly, reduction in nasal or lung viral loads post challenge did not correlate with binding to a single epitope in both species, pointing at strong advantage of broad anti-G antibody responses, generated by the unglycosylated REG. In our recent study in humans, age-dependent antibody titers against the peptide containing the CCD domain (peptide 169–207) was observed. The highest titers were seen in young children (<2 yr) with a clear decline in adults. Binding to peptide 148–178 that contain the epitope targeted by the protective monoclonal antibody 131-2G was also measured in all age groups following natural exposure to RSV infection^8^.

Most earlier studies of vaccine candidates based on the G protein focused on the CCD motif^34^,^35^. However, evaluation of sera from children following primary RSV infection, using whole genome fragment phage display libraries (GFPDL), identified broad anti-G antibody reactivity against diverse antigenic sites of the RSV G glycoprotein^8^ in agreement with the broad diversity of antibody responses in cotton rats following REG vaccination.

Previous study in mice using synthetic peptides spanning the CCD (aa 171–201) or shorter G-segments upstream (aa 67–147) and downstream of the CCD (aa 199–298)^38^ concluded that antibodies against the conserved CCD were most important in protecting mice from viral replication and enhanced lung pathology, possibly due to blocking of CX3C-CX3CR1^38^. In our study, the entire G ectodomain was used for vaccination. We found strong antibody responses to multiple regions of the G protein that correlated with control of viral loads. Therefore, our findings in the cotton rat model support the use of the entire G ectodomain rather than shorter peptides for development of RSV-G based vaccine.

Enhanced lung pathology was previously described in infants and animals vaccinated with the formaldehyde inactivated RSV (FI-RSV) vaccine^15^,^17^,^18^,^19^,^39^. In cotton rats, purified F glycoprotein and chimeric F/G protein produced in baculovirus vector (Bac-FG) did not provide protection due to very low virus-neutralizing antibody titers, similar to FI-RSV. In addition, the vaccinated animals developed high levels of bronchiolar and alveolar histopathology after RSV infection^23^. However, more recently, a number of studies have demonstrated that purified F, particularly in the prefusion conformation but also in the postfusion conformation is capable of inducing high levels of neutralizing and protective antibodies without enhanced pathology^40^. In our early study, mice vaccinated with the fully glycosylated RMG protein resulted in enhanced lung pathology, increased cellular infiltrates (neutrophils, eosinophils) and Th2/Th1 cytokine imbalance^9^, in agreement with other studies using glycosylated G protein expressed in mammalian cells or in recombinant vaccinia virus vector^41^,^42^. In the current preclinical study, enhanced lung pathology following RSV-A2 viral challenge was only observed in animals vaccinated with FI-RSV. The adjuvanted-REG vaccinated animals showed complete protection from viral replication and no lung pathology, similar to the experimentally live RSV-A2 infected group. Importantly, animals vaccinated with the unadjuvanted REG had lower anti-G antibody titers and only partially controlled virus replication in the lungs (Fig. 3B). Yet, this group did not show enhanced lung pathology compared with the PBS sham-vaccinated animals. These findings are in agreement with the lack of enhanced lung pathology in mice vaccinated with REG compared with the fully glycosylated RMG protein as previously described^9^.

Enhanced lung pathology after FI-RSV was reportedly associated with cytokine imbalance and particularly increase in Th2/Th1 cytokine ratio^19^. In our mouse study, increase in IL-4, IL-4/IFN-γ protein ratio, and in several chemokines were only observed in animals with enhanced lung pathology following RMG vaccination and not following REG vaccination^9^. For cotton rats, reagents are not available for quantitation of cytokine proteins in the lungs. However we did find an increase in the levels of IL-4 mRNA and MCP-1 mRNA in the lungs of FI-RSV vaccinated animals at 5 days post challenge that correlated with enhanced lung pathology. In contrast, no increase in IL-4, IFN-γ, or MCP-1 was observed in the lungs of REG-vaccinated animals, correlating with lack of lung pathology.

The underlying mechanisms responsible for the difference in VAERD among different vaccine candidates are not fully understood. Enhanced Th2 cytokines, IL4R expression on CD4^+^ T cells, and enhanced lung disease were also found in mice that were exposed to natural RSV infection as neonates (5 day old), but not in mature animals, following a second challenge^43^. In the case of G protein, it is possible that O-linked sugars characteristic of mammalian cell-expressed glycosylated RMG as well as virion associated G proteins are strong inducers of Th2 cytokines. A role for processing by different glycosylation-specific antigen presenting cell subsets could influence the subsequent balance between Th1 and Th2 cytokines and should be further investigated in both animals and humans^44^,^45^. It seems that in most cases of vaccination associated enhance disease a lack (or very low) neutralizing antibody titers along with high ratio of Th2/Th1 cytokine secreting CD4 cells was observed. Therefore, both parameters should be considered when selecting new vaccine candidates into human trials, especially in the very young population.

Many of the current RSV vaccine candidates contain only subunit F protein. However, several earlier studies have demonstrated the importance for inclusion of both F and G proteins in RSV vaccines^46^,^47^. The results of our current study in cotton rats provide additional strong justification for including G protein in future RSV vaccines. Bacterially produced unglycosylated G protein could provide an economical, safe and effective broad protective vaccine against RSV disease in the very young and aging human population.

## Material and Methods

### Cell, Viruses and Plasmids

A549 cells (CCL-185) were obtained from the ATCC and grown in F12K medium supplemented with 10% heat inactivated fetal bovine serum (FBS) and 1X Penicillin/Streptomycin (P/S) and L-glutamine and maintained in an incubator at 37 °C and 5% CO_2_.

RSV virus strain A2 (NR-12149) and B1 (NR-4052) used for PRNT testing were obtained from BEI Resources, NIAID, NIH and expanded in the laboratory by passage on sub-confluent A549 cell monolayers in F12K media with L-glutamine supplemented with 2% FBS and 1X P/S (infection media).

Codon optimized RSV-G coding DNA for *E. coli* were chemically synthesized. A *Not*I and *Pac*I site was used for cloning the RSV A2 G ectodomain coding sequence (67–298) in the T7 based pSK expression vector to express G protein in *E. coli*.

### Production of Recombinant *E. coli* produced G (REG)

Recombinant RSV G extracellular domain (residues 67–298) was expressed in *E. coli* BL21(DE3) cells (Novagen) and was purified as described previously^9^. Briefly, G protein expressed and localized in *E. coli* inclusion bodies (IB) was isolated by cell lysis and multiple washing steps with 1% Triton X-100. The pelleted IB containing G protein was resuspended in denaturation buffer and centrifuged to remove debris. The protein supernatant was renatured by slowly diluting the redox folding buffer and dialyzed to remove the denaturing agents. The dialysate was filtered through a 0.45 μm filter and was purified through a HisTrap FF chromatography column (GE Healthcare). Protein concentration was analyzed by BCA (Pierce) and purity of the REG protein was determined by SDS-PAGE. Endotoxin levels of the purified protein were <1 EU/μg of protein.

### Plaque reduction neutralization test (PRNT)

For PRNT, heat-inactivated sera was diluted 4-fold and incubated with 20–60 pfu of RSV strains A2 or B1 for 1 hr at 37 °C and 5% CO_2_ and the assay was performed in presence of 5% Guinea Pig complement as described previously^48^. Briefly, A549 cells were infected with sera:virus mix and incubated for 1 hr before removing the inoculum and adding an overlay of 0.8% methylcellulose in infection media. Plates were incubated for 5–7 days and plaques were detected by immunostaining. Neutralization titers were calculated by adding a trend-line to the neutralization curves and using the following formula to calculate 50% end-point titers: antilog of [(50 + y-intercept)/slope].

### Cotton Rat immunization, RSV challenge and sample collection

Inbred female *Sigmodon hispidus* cotton rats between 6 and 8 weeks of age (Source: Sigmovir Biosystems, Inc., Rockville MD) were maintained and handled under veterinary supervision in accordance with the National Institutes of Health guidelines and following an animal study proposal approved by Sigmovir Biosystem’s Institutional Animal Care and Use Committee (IACUC Protocol #15). Cotton rats were housed in clear polycarbonate cages individually. Cotton rat immunization and challenge study, including post-challenge evaluations of viral loads and lung histopathology, were conducted at Sigmovir Biosystems under the NIAID Non-Clinical Evaluation Agreement #HHSN272201000006I/HHSN27200011.

The prototype A2 strain of RSV (ATCC, Manassas, VA) for challenge of cotton rats was propagated in HEp-2 cells and subjected to serial plaque-purification to reduce defective-interfering particles. A stock of virus containing approximately 3.0 × 10^8^ pfu/ml in sucrose stabilizing media was used for this *in vivo* experiment. This stock of virus is stored at −80 °C and has been characterized *in vivo* using the cotton rat model and validated for upper and lower respiratory tract replication.

Fifty five (55) female cotton rats 6–8 weeks of age were divided into 5 groups A thru E as indicated in the table in Fig. 1A. A baseline serum sample was obtained from all animals followed by intramuscular immunization (IM, quadriceps) with different vaccines (Groups A-E) on day 0 and day 28. Cotton rats in Group F were inoculated intranasally (IN) with 0.1 ml of PBS containing 10^5 pfu of live RSV strain A2 (experimental infection).

PBS (pH 7.4 w/o Ca++ or Mg++) was used as mock control (Groups A and B), and for dilution of vaccine formulations. For preparation of adjuvanted vaccine, Recombinant RSV G protein (RSV-G) was diluted to 100 μg/ml in PBS and mixed in 1:1 ratio (by volume) with Emulsigen adjuvant (MVP Technologies) using the 3-way valve connected to two luer lock syringes to a final protein concentration of 50 μg/ml. Nonadjuvanted RSV-G vaccine was prepared by diluting RSV-G to 50 μg/ml using PBS. FI-RSV Lot #100, produced in the mid-1960s by Pfizer Inc. and stored at 4 °C, was diluted 1:100 using PBS. All formulations at 0.1 ml were injected within 30 minutes of preparation.

Blood was collected by eye bleed on days 0, 28 and 49. On day 49, group A was mock challenge intranasally (i.n.) with 0.1 ml of PBS (pH 7.4). All other groups (B-F) were challenged intranasally with 0.1 ml of RSV/A2 at 10^5^ pfu per animal.

On Day 2 and Day 5 post-virus challenge, 5 animals from groups B thru F were sacrificed. Nasal tissues and lungs were harvested and homogenized in 3 ml of HBSS supplemented with 10% SPG for viral titrations. Lung tissues were also flash frozen in liquid nitrogen for subsequent cytokine qPCR or inflated with 10% neutral buffered formalin for histopathology (Day 5 only).

### Lung and nose viral load determination

Lung and nose homogenates were clarified by centrifugation and diluted in EMEM. Confluent HEp-2 monolayers were infected in duplicate with diluted homogenates in 24 well plates. After one hour incubation at 37 °C in a 5% CO_2_ incubator, the wells were overlayed with 0.75% Methylcellulose medium. After 4 days of incubation, the overlay was removed and the cells were fixed with 0.1% crystal violet stain for one hour and then rinsed and air dried. Plaques were counted and virus titers (plaque forming units per gram of tissue) were calculated as geometric mean ± standard error for all animals in a group at a given time.

### Measurement of cytokine levels in the lung by Real-time PCR

Total RNA was extracted from homogenized lung tissues using the RNeasy purification kit (Qiagen). One μg of total RNA was used to prepare cDNA using Super Script II RT (Invitrogen) and used for the real-time PCR reactions using Bio-Rad iQTM SYBR Green Supermix. Reactions were set up in duplicates in 96-well trays and amplifications were performed on a Bio-Rad iCycler. The baseline cycles and cycle threshold (Ct) were calculated by the iQ5 software in the PCR Base Line Subtracted Curve Fit mode. Relative quantitation of DNA was applied to all samples. Standard curves were developed using serially diluted cDNA sample most enriched in the transcript of interest. The Ct values are plotted against log10 cDNA dilution factor. These curves were used to convert the Ct values obtained for different samples to relative expression units. These relative expression units were then normalized to the level of β-actin mRNA expressed in the corresponding samples. For animal studies, mRNA levels are expressed as the geometric mean ± SEM for all animals in a group at a given time.

### Pulmonary histopathology

Lungs were dissected and inflated with 10% neutral buffered formalin to their normal volume, and then immersed in the same fixative solution. Following fixation, lungs were embedded in paraffin, sectioned and stained with hematoxylin and eosin (H&E). Four parameters of pulmonary inflammation were evaluated: peribronchiolitis (inflammatory cell infiltration around the bronchioles), perivasculitis (inflammatory cell infiltration around the small blood vessels), interstitial pneumonia (inflammatory cell infiltration and thickening of alveolar walls), and alveolitis (cells within the alveolar spaces). Slides were scored blindly on a 0–4 severity scale and were subsequently converted to a 0–100% histopathology scale.

### Surface Plasmon Resonance (SPR)

Steady-state equilibrium binding of post-vaccination sera was monitored at 25 °C using a ProteOn surface plasmon resonance biosensor (BioRad). The recombinant RSV G proteins from *E. coli* (REG) or from mammalian 293 T cells (RMG; Recombinant Mammalian cell derived G) were coupled to a GLC sensor chip via amine coupling with 500 resonance units (RU) in the test flow channels. For polyclonal post-vaccination serum antibody kinetics against individual RSV-G peptides, the SPR analysis was performed using peptides biotinylated at C-terminus captured on a NLC chip surface. Samples of 100 μl freshly prepared sera dilutions were injected at a flow rate of 50 μl/min (120 sec contact duration) for association, and disassociation was performed over a 600 second interval. Responses from the protein surface were corrected for the response from a mock surface and for responses from a buffer only injection. Pre-vaccination cotton rat sera and a IgG depleted sera were used as a negative control. Total antibody binding and data analysis results were calculated with BioRad ProteOn manager software (version 3.1).

### Statistical Analyses

The statistical significances of group differences were determined using a one-way ANOVA and Bonferroni multiple comparisons test. Correlations were calculated with a Spearman’s two-tailed test. p-values less than 0.05 were considered significant with a 95% confidence interval.

## Additional Information

**How to cite this article:** Fuentes, S. *et al*. Preclinical evaluation of bacterially produced RSV-G protein vaccine: Strong protection against RSV challenge in cotton rat model. *Sci. Rep.* **7**, 42428; doi: 10.1038/srep42428 (2017).

**Publisher's note:** Springer Nature remains neutral with regard to jurisdictional claims in published maps and institutional affiliations.
